# Subjective sleep quality and fatigue assessment in Polish adult patients with primary immunodeficiencies: A pilot study

**DOI:** 10.3389/fimmu.2022.1028890

**Published:** 2023-01-13

**Authors:** Kinga Grochowalska, Marcin Ziętkiewicz, Ewa Więsik-Szewczyk, Aleksandra Matyja-Bednarczyk, Katarzyna Napiórkowska-Baran, Katarzyna Nowicka-Sauer, Adam Hajduk, Dariusz Sołdacki, Zbigniew Zdrojewski

**Affiliations:** ^1^ Department of Rheumatology, Clinical Immunology, Geriatrics and Internal Medicine, Medical University of Gdańsk, Gdańsk, Poland; ^2^ Department of Internal Medicine, Pneumonology, Allergology and Clinical Immunology, Central Clinical Hospital of the Ministry of National Defense, Military Institute of Medicine - National Research Institute, Warsaw, Poland; ^3^ Outpatient Clinic for the Immunological Hypercoagulable Diseases, The University Hospital in Krakow, Kraków, Poland; ^4^ Department of Allergology, Clinical Immunology and Internal Diseases, Ludwik Rydygier Collegium Medicum in Bydgoszcz Nicolaus Copernicus University in Torun, Bydgoszcz, Poland; ^5^ Department of Family Medicine, Medical University of Gdańsk, Gdańsk, Poland

**Keywords:** sleep quality, fatigue assessment, primary immunodeficiencies, heterogeneous disorders, polish, inborn errors of immunity

## Abstract

**Introduction:**

Primary immunodeficiencies (PIDs) are clinically heterogeneous disorders caused by abnormalities in the immune system. However, PIDs are genetically determined and may occur at any age from early childhood to elderly age. Due to chronic patterns, the risk of malignancy and organ damage in patients with PIDs may affect any aspect of life, including sleep patterns. To our knowledge, the prevalence of insomnia and subjective sleep quality have not been investigated in patients with PIDs. Therefore, this pilot study was conducted to investigate sleep quality, the prevalence of sleep disturbances, and fatigue in adult patients with PIDs in Poland.

**Methods:**

All participants were surveyed using the Athens Insomnia Scale, Pittsburgh Sleep Quality Index, Fatigue Severity Scale, and a questionnaire concerning general health and demographic data. We included 92 participants: 48 women (52.2%) and 44 men (47.8%).

**Results:**

Participants’ mean age was 41.9 ± 13.9 years. The mean sleep duration was 7.0 ± 1.5 hours, and the mean sleep latency was 41.2 ± 53.1 minutes. Additionally, 44.6% of patients (n=41) had symptoms of insomnia and 44.6% (n=42) had poor sleep quality. Less than one-fourth (n=22; 23.9%) of the patients reported the use of sleeping pills; moreover, clinically significant fatigue was reported in 52.2% (n=48).

**Discussion:**

Our investigation provides insight into the problem of sleep disturbances in patients with PIDs. Data have demonstrated that sleeping disorders with concomitant fatigue are common in patients with PID. Further studies are needed to determine the determinants of poor sleep quality in this specific group of patients.

## 1 Introduction

Primary immunodeficiencies (PIDs) are clinically heterogeneous group of disorders caused by abnormalities in the innate immune system. The onset of the disease occurs mostly in childhood; however, the initial symptoms may appear at any age (1). Based on national registers, the prevalence of symptomatic PIDs varies from 1:8500 to 1:100000 (2).

Clinical manifestations of PIDs include recurrent bacterial infections of the upper and lower respiratory and gastrointestinal tracts, as well as meningitis, arthritis, and skin and organ abscesses (3). Infections are characterized by a severe course and cannot always be treated with standard medications (4). As a consequence of recurrent respiratory system infections, patients develop bronchiectasis, chronic obstructive pulmonary disease (COPD), and interstitial lung disease (5).Viral infections of the respiratory tract, gastrointestinal tract, and skin are also common (6).

Furthermore, genetic defects lead to atopy, multi-organ autoimmunization, lymphoproliferation, and vulnerability to neoplastic and autoinflammatory diseases (1). The symptoms of autoimmunization may precede the occurrence of infection (7, 8). Due to chronic course, the risk of malignancy, and organ damage, PIDs may affect any aspect of patients’ lives, including sleep patterns and quality of life.

Sleep is essential to humans. Sleep provides physical restoration (9), promotes memory consolidation (10), and maintains proper function of the immune system (11); however, its exact role remains unknown. The International Classification of Sleep Disorders has identified 7 major categories of sleep disorders: insomnia disorders, sleep-related breathing disorders, central disorders of hypersomnolence, circadian rhythm sleep-wake disorders, sleep-related movement disorders, parasomnias, and other sleep disorders (12).

Fatigue is tiredness or weakness experienced by healthy individuals in certain situations and resolve with resting. Aggravated fatigue that limits daily functioning is considered a deviation from the norm. When fatigue lasts more than 6 months, it is referred to as chronic fatigue, with prevalence in the general population varying from 13 to 30% (13, 14). It is more common in patients with chronic diseases, with the highest prevalence in those with autoimmune disorders (15). Although the association between various chronic diseases and fatigue has been highlighted in many studies, the underlying mechanism remains unclear.

Various sleep disorders have been investigated in chronic diseases, including rheumatic diseases (16), autoimmune diseases (15), lung diseases (17), and cardiovascular diseases (18). However, to the best of our knowledge, the prevalence of insomnia and subjective sleep quality have not been investigated in patients with PIDs. To fill this gap, we conducted a pilot study focusing on sleep quality and prevalence of insomnia in patients with PID.

## 2 Materials and methods

### 2.1 Study design

**Figure 1:**
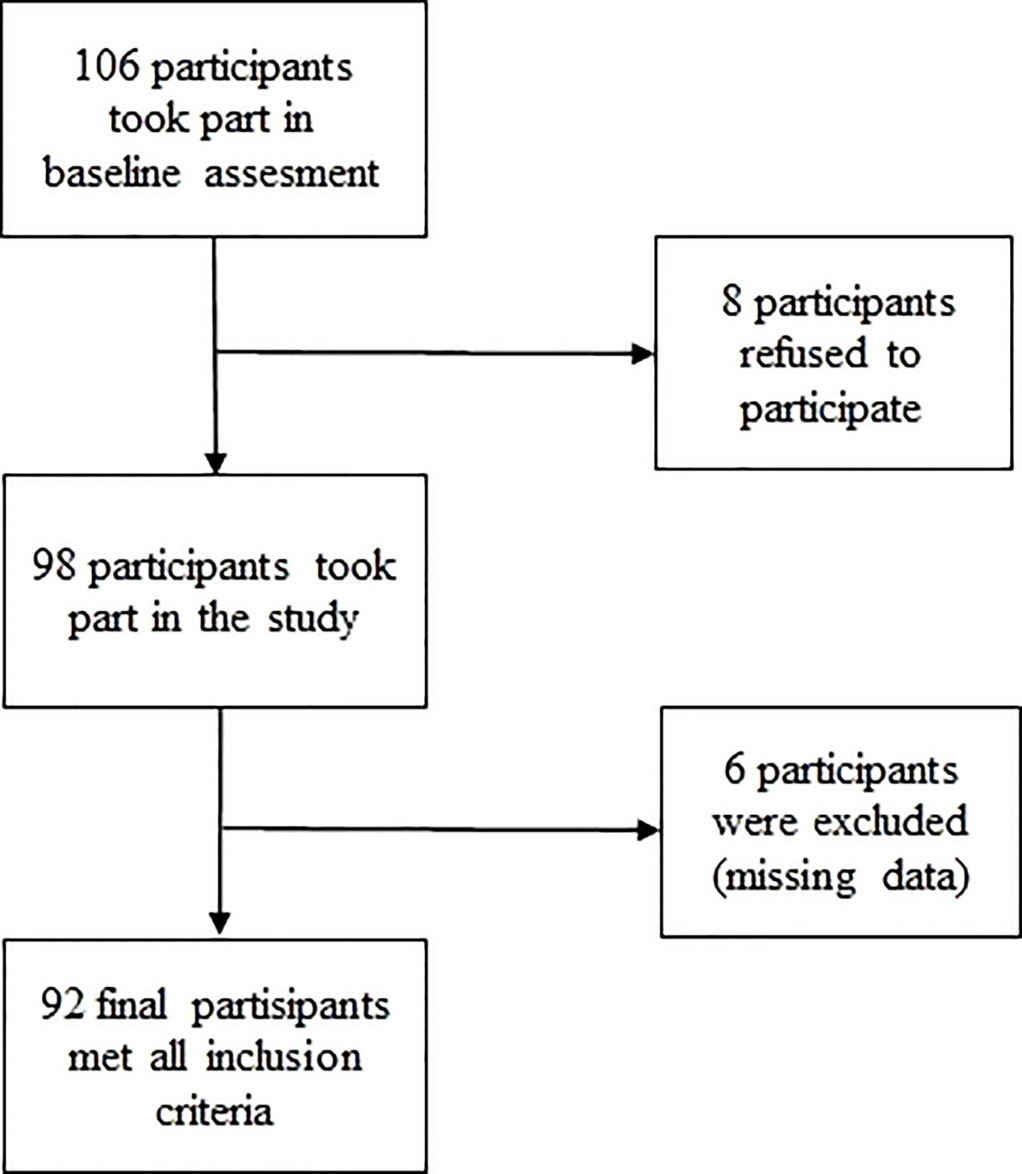
Flow chart of patients’ selection.

This pilot study investigated sleep characteristics, the prevalence of insomnia, subjective sleep quality, and fatigue in adult patients with PIDs in Poland. The study was conducted from February 2021 to February 2022 at 4 Polish clinical centers in Bydgoszcz, Gdańsk, Kraków, and Warszawa. The inclusion criteria were as follows: age ≥18 years, diagnosis of PIDs according to the diagnostic criteria of the European Society for Immunodeficiencies (19), and written consent. Patients who did not meet the inclusion criteria, did not agree to participate in the study, or did not complete their questionnaires were excluded from the study (**Figure 1**).

Data were collected using questionnaires. All participants were surveyed using the following scales and questionnaires: the Athens Insomnia Scale (AIS), Pittsburgh Sleep Quality Index (PSQI), and Fatigue Severity Scale (FSS). The survey included demographic questions to collect data on age, sex, work, residential status, comorbidities, PID-related factors, and type of immunoglobulin replacement therapy. Additionally, we assessed anxiety and depression using the Hospital Anxiety and Depression Scale (HADS).

### 2.2 Athens insomnia scale

The AIS is a self-report questionnaire used to assess the severity of insomnia based on the diagnostic criteria of the International Classification of Diseases (ICD), Tenth Revision. It comprises 8 items with a score of 0 to 3 for each item, where 0 indicates no problem at all and 3 indicates a very serious problem (20). According to the authors of the AIS, the cut-off value for insomnia is 6 points; however, some researchers use 8 points as the cut-off value. According to the validation of the Polish version of the AIS, we assumed that a global score ≥8 indicated insomnia. The psychometric properties of the Polish version of the AIS are highly satisfactory (Cronbach’s alpha, 0.90) (21). In our study, Cronbach’s alpha was 0.879.

### 2.3 Pittsburgh sleep quality index

The PSQI is used to measure self-reported sleep quality and sleep disturbances over the previous month. Nineteen items were evaluated with a score of 0–3, and they constituted 7 components: subjective sleep quality, sleep latency, sleep duration, habitual sleep efficiency, sleep disturbances, use of sleeping medication, and daytime dysfunction. The global score (range from 0 to 21), which constitutes the sum of the scores for the 7 components, indicates sleep quality. A global score ≤5 is associated with good sleep quality, whereas a global score >5 is associated with poor sleep quality (22). Internal consistency measured with Cronbach’s alpha was 0.803.

### 2.4 Fatigue severity scale

The FSS is a self-reported 9-item questionnaire for measuring fatigue. Each item is evaluated with scores ranging from 1 to 7, where 1 corresponds to strong disagreement and 7 corresponds to strong agreement. The mean score of the items was used as the FSS score, with a score ≥4 indicating fatigue (23). Psychometric properties of the Polish version of the FSS were satisfactory (Cronbach’s alpha, 0.915)

### 2.5 Hospital anxiety and depression scale

The HADS comprises 14 questions, 7 for each subscale, rated using a 0 to 3 response Likert scale (24). The maximum score for each subscale is 21 points. The cut-off value for moderate anxiety or depression is ≥8, while that for severe depressive or anxiety symptoms is ≥11 points. Scores below 8 indicate a normal result (25).

### 2.6 Statistical analysis

The normality of the observed values was tested using the Shapiro–Wilk test. Continuous variables were analyzed using the Student t-, Mann–Whitney U, and Kruskal–Wallis tests. Categorical variables were analyzed using the chi-square or Fisher exact test. Data were also assessed using Pearson or Spearman correlation analysis to estimate correlations between the variables. Multiple linear regression analyses were performed to investigate the predictors of AIS total score, FSS total score, and PSQI total score. For all data analyses, differences were considered statistically significant at *p*<0.05. Statistical analysis was performed using STATISTICA software (version 13; TIBCO Software Inc., Palo Alto, CA, USA).

## 3 Results

### 3.1 Study population characteristics

A total of 106 individuals took part in the baseline assessment. Eight participants refused to participate. Six individuals were excluded because they did not complete the questionnaire. Finally, the study included 92 participants: 48 women (52.2%) and 44 men (47.8%) **(** **Figure 1** **).** Participants’ mean age was 41.9 ± 13.9 years. Most patients lived in cities (n=66; 71.7%). Almost half of the participants had higher education (n=43, 46.7%), 37.0% (n=34) had secondary education, and 16.3% (n=15) had primary or vocational education. Sixty patients (65.3%) had regular employment.

Common variable immunodeficiency (CVID) was the most frequent PID in our study group (n=47; 51.1%). Immunoglobulin G subclass deficiency affected 18.5% of patients (n=17), X-linked agammaglobulinemia affected 9.8% of patients (n=9), and other humoral immunodeficiencies affected 16.3% (n=15). We categorized the remaining 4.3% of patients (n=4) as having other PIDs. Most patients received immunoglobulin replacement therapy (n=87; 94.4%). In this group, subcutaneous immunoglobulins were administered to 85.1% of patients (n=74), and intravenous immunoglobulins were administered to 14.9% (n=13). The mean diagnostic delay of PIDs was 7.2 ± 10.8 years.

The majority of patients (66.3%; n=61) had comorbidities. Seventeen patients (27.9%) had one chronic disease, 15 (24.6%) had two chronic diseases, and 29 (47.5%) had three chronic diseases. Neoplastic disease affected 12 patients (13.0%). Two patients underwent ongoing cancer treatment. The remaining 10 patients were survivors of cancer. Among them, 7 patients had a history of lymphoma, and 3 other patients had histories of lung cancer, thyroid cancer, and carcinoid. We also assessed additional factors that could have influenced participants’ sleep patterns. Depression was declared as a comorbidity by 7.6% of participants (n=7). A stressful event in the last 3 months was declared by 54.3% (n=50) and was mainly negative (n=42; 84.0%). General pain was present in 75 patients (81.5%), with daily occurrence in the last 3 months in 29 of them (38.7%). Only 17 patients (18.5%) did not report pain in the last 3 months. Addictions were declared by 15.2% (n=14) of patients, of whom 12 had a nicotine addiction, 2 patients were addicted to medications, and none declared a narcotic addiction.

### 3.2 Subjective sleep quality and symptoms of insomnia

The mean sleep duration declared by patients was 7.0 ± 1.5 hours (range, 3–11 hours), and approximately one-third of them slept <7 hours (n=30; 32.6%). Sleep latency lasted ≤30 minutes in 68 patients (73.0%) with a mean value of 41.2 ± 53.1 minutes (range, 2–300 minutes) **(** **Table 1** **)**. Almost half of the patients had an AIS total score ≥8 (n=41; 44.6%) and PSQI total score >5 (n=42; 45.7%). The mean AIS was 7.9 ± 5.2 **(** **Table 2** **)**, and the mean PSQI was 6.4 ± 4.1.

**Table 1:** Components of PSQI (0 – 3 p.).

Variable	Mean ± SD
Sleep efficiency	0.6 ± 0.9
Duration of sleep	0.6 ± 0.9
Sleep latency	1.4 ± 1.0
Sleep disturbance	1.3 ± 0.6
Overall sleep quality	1.3 ± 0.8
Need meds to sleep	0.5 ± 1.0
Day dysfunction due to sleepiness	1.0 ± 0.9
**PSQI Total Score (0 – 21 p.)**	**6.4 ± 4.1**
Sleep latency (min)	41.2 ± 53.1
Sleep duration (h)	7.0 ± 1.5

PSQI, Pittsburgh Sleep Quality Index; SD, standard deviation; min, minutes; h, hours; p, point.

**Table 2:** Components of AIS (0 – 3 p.).

Variable	Mean ± SD
Sleep induction	1.4 ± 1.1
Awakenings during the night	1.4 ± 0.8
Final awakening earlier than desired	0.9 ± 0.9
Overall sleep duration	0.8 ± 0.9
Overall quality of sleep	0.9 ± 0.9
Sense of well-being during the day	0.81 ± 0.9
Functioning suring the day	0.7 ± 0.7
Sleepiness during the day	1.1 ± 0.7
**AIS total score (0 – 24 p.)**	**7.9 ± 5.2**

AIS, Athens Insomnia Scale; SD, Standard deviation; p, point.

Participants’ mean age was comparable between those with and without insomnia (42.3 ± 14.6 and 41.6 ± 13.4, p=0.827, respectively). Patients with poor sleep quality were older (44.6 ± 14.8) than those with good sleep quality (39.7 ± 12.8); however, the difference was not statistically significant (p=0.089).

Women had poor sleep quality more frequently (n=28; 58.3%) than men did (n=14; 31.8%, p=0.013). Patients with poor sleep quality had a higher body mass index (BMI) than those with good sleep quality (25.4 ± 4.9 vs. 23.2 ± 4.6, p=0.026) **(** **Table 3** **)**. Symptoms of insomnia and subjective sleep quality were not associated with demographic characteristics, such as age, education, regular work status, or residential status **(** **Table 4** **)**.

**Table 3:** Comparison of patients with good or poor sleep quality (PSQI), absence or presence of insomnia symptoms, and absence or presence of fatigue symptoms according to sociodemographic data.

	Sleep quality (PSQI)		Insomnia symptoms (AIS)		Fatigue (FSS)	
	good	poor	absence	presence	absence	presence
number (%)	50 (54.3%)	42 (45.7%)	51 (55.4%)	41 (44.6%)	44 (47.8%)	48 (52.2%)
**Age** (mean ± SD)	39.7 ± 12.8	44.6 ± 14.8	41.6 ± 13.4	42.3 ± 14.6	39.7 ± 13.5	43.9 ± 14.0
Sex, number (%)						
Female	20 (41.7)	28 (58.3)*	22 (45.8)	26 (54.2)	18 (37.5)	30 (62.5)
Male	30 (68.2)	14 (31.8)*	29 (65.9)	15 (34.1)	26 (59.1)	18 (40.9)
Education n (%)						
Primary or vocational	9 (60.0)	6 (40.0)	9 (60.0)	6 (40.0)	9 (60.0)	6 (40.0)
Higher	24 (55.8)	19 (44.2)	22 (51.2)	21 (48.8)	18 (41.9)	25 (58.1)
Secondary	17 (50.0)	17 (50.0)	20 (58.8)	14 (41.2)	17 (50.0)	17 (50.0)
Work status n (%)						
Unemployed	0 (0.0)	4 (100.0)	1 (25.0)	3 (75.0)	0 (0.0)	4 (100.0)
Retiree	2 (33.3)	4 (66.7)	2 (33.3)	4 (66.7)	2 (33.3)	4 (66.7)
Physical worker	8 (72.7)	3 (27.3)	7 (63.6)	4 (36.4)	9 (81.8)	2 (18.2)
Office-worker	22 (53.7)	19 (46.3)	24 (58.5)	17 (41.5)	20 (48.8)	21 (51.2)
Annuitant	13 (59.1)	9 (40.9)	13 (59.1)	9 (40.9)	9 (40.9)	13 (59.1)
Student	5 (62.5)	3 (37.5)	4 (50.0)	4 (50.0)	4 (50.0)	4 (50.0)
Residential status n (%)						
Village	18 (69.2)	8 (30.8)	18 (69.2)	8 (30.8)	14 (53.8)	12 (46.2)
City ≤ 50 000 habitants	6 (35.3)	11 (64.7)	6 (35.3)	11 (64.7)	6 (35.3)	11 (64.7)
City 50 000 -100 000 habitants	9 (69.2)	4 (30.8)	9 (69.2)	4 (30.8)	7 (53.8)	6 (46.2)
City ≥100 000 habitants	17 (47.2)	19 (52.8)	18 (50.0)	18 (50.0)	17 (47.2)	19 (52.8)

PSQI, Pittsburgh Sleep Quality Index; SD, standard deviation; AIS, Athens Insomnia Scale; FSS, Fatigue Severity Scale; BMI (kg/m^2^), body mass index; *p <.05.

**Table 4:** Comparison of patients with good or poor sleep quality (PSQI), absence or presence of insomnia symptoms, and absence or presence of fatigue symptoms according to clinical data.

	Sleep quality (PSQI)		Insomnia symptoms (AIS)		Fatigue (FSS)	
	good	poor	absence	presence	absence	presence
number (%)	50 (54.3%)	42 (45.7%)	51 (55.4%)	41 (44.6%)	44 (47.8%)	48 (52.2%)
Body Mass Index (BMI, kg/m^2^)						
(mean ± SD)	23.2 ± 4.6*	225.4 ± 4.9*	23.9 ± 4.7	24.7 ± 5.1	22.9 ± 4.9	25.4 ± 4.6*
Presence of chronic disease (other than PIDs) n(%)						
No	20 (66.7)	10 (33.3)	19 (63.3)	11 (36.7)	17 (56.7)	13 (43.3)
Yes	30 (48.4)	32 (51.6)	32 (51.6)	30 (48.4)	27 (43.5)	35 (56.5)
Number of chronic diseases						
(mean ± SD)	1.0 ± 1.3	2.0 ± 2.9***	1.0 ± 1.9	2.0 ± 2.8*	1.0 ± 2.0	2.0 ± 2.7*
Autoimmune phenomena n (%)						
No	41 (63.1)	24 (36.9)*	37 (56.9)	28 (43.1)	36 (55.4)	29 (44.6)*
Yes	9 (33.3)	18 (66.7)*	14 (51.9)	13 (48.1)	8 (29.6)	19 (70.4)*
Depression n (%)						
No	49 (57.6)	36 (42.4)*	48 (56.5)	37 (43.5)	43 (50.6)	42 (49.4)
Yes	1 (14.3)	6 (85.7)*	3 (42.9)	4 (57.1)	1 (14.3)	6 (85.7)
Neoplastic disease n (%)						
No	47 (58.8)	33 (41.2)*	47 (58.8)	33 (41.2)	42 (52.5)	38 (47.5)*
Yes	3 (25.0)	9 (75.0)*	4 (33.3)	8 (66.7)	2 (16.7)	10 (83.3)*
Addiction n (%)						
No	46 (59.0)	32 (41.0)*	46 (59.0)	32 (41.0)	38 (48.7)	40 (51.3)
Yes	4 (28.6)	10 (71.4)*	5 (35.7)	9 (64.3)	6 (42.9)	8 (57.1)
Active smoker n (%)						
No	47 (58.8)	33 (41.2)*	47 (58.8)	33 (41.2)	39 (48.8)	41 (51.2)
Yes	3 (25.0)	9 (75.0)*	4 (33.3)	8 (66.7)	5 (41.7)	7 (58.3)
Presence of pain in previous 3 months n (%)						
No	14 (82.4)	3 (17.6)*	13 (76.5)	4 (23.5)	12 (70.6)	5 (29.4)
Yes	36 (48.0)	39 (52.0)*	38 (50.7)	37 (49.3)	32 (42.7)	43 (57.3)
Frequency of general pain in previous 3 months n (%)						
Almost everyday	7 (24.1)	22 (75.9)***	9 (31.0)	20 (69.0)**	8 (27.6)	21 (72.4)**
For several days	27 (65.9)	14 (34.1)***	27 (65.9)	14 (34.1)**	24 (58.5)	17 (41.5)**
For more than 30 days	2 (40.0)	3 (60.0)***	2 (40.0)	3 (60.0)**	0 (0.0)	5 (100.0)**
Not at all	14 (82.4)	3 (17.6)***	13 (76.5)	4 (23.5)**	12 (70.6)	5 (29.4)**
The stressful event in the previous 3 months n (%)						
No	28 (66.7)	14 (33.3)*	28 (66.7)	14 (33.3)	23 (54.8)	19 (45.2)
Yes	22 (44.0)	28 (56.0)*	23 (46.0)	27 (54.0)	21 (42.0)	29 (58.0)
The character of a stressful event n (%)						
Negative	15 (35.7)	27 (64.3)***	16 (38.1)	26 (61.9)**	18 (42.9)	24 (57.1)
Positive	8 (100.0)	0 (0.0)***	8 (100.0)	0 (0.0)**	4 (50.0)	4 (50.0)
Sleeping medications administration; n (%)						
No	47 (67.1)	23 (32.9)***	45 (64.3)	25 (35.7)**	37 (52.9)	33 (47.1)
Yes	3 (13.6)	19 (86.4)***	6 (27.3)	16 (72.7)**	7 (31.8)	15 (68.2)

PSQI, Pittsburgh Sleep Quality Index; SD, standard deviation; AIS, Athens Insomnia Scale; FSS, Fatigue Severity Scale; *p <.05. **p <.01. ***p <.001.

Patients who had poor sleep quality had a higher number of chronic diseases than those with good sleep quality (2.0 ± 2.9 vs. 1.0 ± 1.3, p<0.001). Likewise, patients with insomnia had more chronic diseases than those without symptoms of insomnia (2.0 ± 2.8 vs. 1.0 ± 1.9, p=0.046). The number of chronic diseases correlated with the AIS total score (r=0.48, p<0.001) and PSQI total score (r=0.45 p<0.001) **(** **Table 5** **)**. Patients who experienced general pain almost every day for 3 months prior to the study had poor sleep quality (n=22, 75.9%; p<0.001) and symptoms of insomnia (n=20, 69.0%; p=0.006) **(** **Table 3** **)**. Intensity of pain was correlated with PSQI total score (r=0.34, p<0.01) and AIS total score (r=0.36, p<0.01) **(** **Table 5** **).**

**Table 5:** Comparison of patients with good or poor sleep quality (PSQI), absence or presence of insomnia symptoms, and absence or presence of fatigue symptoms according to primary immunodeficiencies (PIDs).

		Sleep quality (PSQI)		Insomnia symptoms (AIS)		Fatigue (FSS)	
		good	poor	absence	presence	absence	presence
**number (%)**		50 (54.3%)	42 (45.7%)	51 (55.4%)	41 (44.6%)	44 (47.8%)	48 (52.2%)
PIDs n (%)							
CVID		28 (59.6)	19 (40.4)	24 (51.1)	23 (48.9)	25 (53.2)	22 (46.8)
IgG subclasses deficiency		7 (41.2)	10 (58.8)	9 (52.9)	8 (47.1)	5 (29.4)	12 (70.6)
XLA		5 (55.6)	4 (44.4)	6 (66.7)	3 (33.3)	5 (55.6)	4 (44.4)
other immuno-deficiencies		4 (100.0)	0 (0.0)	2 (50.0)	2 (50.0)	3 (75.0)	1 (25.0)
other humoral immuno-deficiencies		6 (40.0)	9 (60.0)	10 (66.7)	5 (33.3)	6 (40.0)	9 (60.0)
PIDs treatment n (%)							
without treatment		3 (75.0)	1 (25.0)	1 (25.0)	3 (75.0)	3 (75.0)	1 (25.0)
other than IG replacement therapy		0 (0.0)	1 (100.0)	1 (100.0)	0 (0.0)	0 (0.0)	1 (100.0)
IVIG		4 (30.8)	9 (69.2)	7 (53.8)	6 (46.2)	6 (46.2)	7 (53.8)
SCIG		43 (58.1)	31 (41.9)	42 (56.8)	32 (43.2)	35 (47.3)	39 (52.7)
Age of first symptoms of PIDs (years)							
(mean ± SD)		21.3 ± 18.4	24.3 ± 18.7	22.4 ± 19.9	22.9 ± 16.9	20.9 ± 18.4	24.2 ± 18.7
Age of PIDs diagnosis (years)							
(mean ± SD)		27.3 ± 16.6	32.9 ± 16.7	29.6 ± 17.6	30.2 ± 15.9	25.8 ± 16.9	33.6 ± 15.9*
Diagnostic delay (years)							
(mean ± SD)		6 ± 8.8	8.6 ± 12.7	7.2 ± 10.3	7.2 ± 11.5	4.9 ± 6.9	9.3 ± 13.1
Immunoglobulin replacement therapy n (%)							
IVIg		4 (30.8)	9 (69.2)	7 (53.8)	6 (46.2)	6 (46.2)	7 (53.8)
SCIg		43 (58.1)	31 (41.9)	42 (56.8)	32 (43.2)	35 (47.3)	39 (52.7)
Place of immunoglobulin administration n (%)							
home		40 (57.1)	30 (42.9)	40 (57.1)	30 (42.9)	33 (47.1)	37 (52.9)
outpatient’s clinic		0 (0.0)	2 (100.0)	1 (50.0)	1 (50.0)	1 (50.0)	1 (50.0)
hospital		7 (46.7)	8 (53.3)	8 (53.3)	7 (46.7)	7 (46.7)	8 (53.3)

PSQI, Pittsburgh Sleep Quality Index; SD, standard deviation; AIS, Athens Insomnia Scale; FSS, Fatigue Severity Scale; PIDs, primary immunodeficiencies; CVID, common variable immunodeficiency; XLA, X-linked agammaglobulinemia; Ig, immunoglobulin; IVIg, intravenous immunoglobin; SCIg, subcutaneous immunoglobulin; *p <.05.

Patients with depression (p=0.044), autoimmune phenomena (p=0.012), cancer survivors (p=0.034), nicotine addicts (p=0.034), and those who experienced stressful events (p=0.037) had poorer sleep quality than their counterparts **(** **Table 3** **)**, and the differences were statistically significant. However, these factors were not related to insomnia.

In the entire group, there was no association between the AIS total score or PSQI total score and the particular PID (p=0.177), diagnostic delay (p=0.846), type of immunoglobulin replacement therapy (p=0.079), location of PID treatment (p=0.229), or hospitalization in the previous 3 months (p=0.578) (**Tables 6**, **7**). Patients with poor sleep quality had statistically more infections 3 months before the study than those with good sleep quality (1.1 ± 1.4 vs. 0.5 ± 0.9, p=0.03). The severity of infection did not affect sleep quality (p=0.534) or the symptoms of insomnia (p=0.055)

**Table 6:** Comparison of patients with good or poor sleep quality (PSQI), absence or presence of insomnia symptoms, and absence or presence of fatigue symptoms according to exacerbation of PID.

		Sleep quality (PSQI)		Insomnia symptoms (AIS)		Fatigue (FSS)	
		good	poor	absence	presence	absence	presence
number (%)		50 (54.3%)	42 (45.7%)	51 (55.4%)	41 (44.6%)	44 (47.8%)	48 (52.2%)
Presence of infections in previous 3 months n (%)							
No		34 (63.0)	20 (37.0)	33 (61.1)	21 (38.9)	27 (50.0)	27 (50.0)
Yes		16 (42.1)	22 (57.9)	18 (47.4)	20 (52.6)	17 (44.7)	21 (55.3)
Number of infections in previous 3 months (mean ± SD)							
		0.5 ± 0.9	1.1 ± 1.4**	0.6 ± 0.9	1.1 ± 1.4	0.7 ± 1.0	0.9 ± 1.3
The severity of infection in the previous 3 months n (%)							
severe		1 (25.0)	3 (75.0)	0 (0.0)	4 (100.0)	0 (0.0)	4 (100.0)
moderate		11 (52.4)	10 (47.6)	13 (61.9)	8 (38.1)	12 (57.1)	9 (42.9)
benign		4 (30.8)	9 (69.2)	5 (38.5)	8 (61.5)	5 (38.5)	8 (61.5)
Administration of antibiotics in previous 3 months n (%)							
No		38 (56.7)	29 (43.3)	38 (56.7)	29 (43.3)	32 (47.8)	35 (52.2)
Yes		12 (48.0)	13 (52.0)	13 (52.0)	12 (48.0)	12 (48.0)	13 (52.0)
Number of antibiotics administrated in previous 3 months (mean ± SD)							
		0.4 ± 0.8	0.5 ± 0.8	0.4 ± 0.7	0.5 ± 0.9	0.4 ± 0.7	0.5 ± 0.9
Hospitalization in previous 3 months (other reason than immunoglobulin administration) n (%)							
No		43 (55.8)	34 (44.2)	46 (59.7)	31 (40.3)	38 (49.4)	39 (50.6)
Yes		7 (46.7)	8 (53.3)	5 (33.3)	10 (66.7)	6 (40.0)	9 (60.0)

PSQI, Pittsburgh Sleep Quality Index; SD, standard deviation; AIS, Athens Insomnia Scale; FSS, Fatigue Severity Scale; PID, primary immunodeficiency; ** p <.01.

**Table 7:** Correlation matrix (Pearson’s r).

	age (years)	BMI (kg/m2)	diagnostic delay (years)	infections in previous 3 months (n)	antibiotics administration in previous 3 months (n)	comorbidities (n)	pain intensity (NRS)	total sleep duration (h)	PSQI total score	AIS total score	FSS total score
**age (years)**	—										
**BMI (kg/m^2^)**	0.36***	—									
**diagnostic delay (years)**	0.14	0.11	—								
**infections in previous 3 months (n)**	0.02	0.21	0.23	—							
**antibiotics administration in previous 3 months (n)**	0.01	0.08	-0.01	0.58***	—						
**comorbidities (n)**	0.47***	0.22	0.19	0.05	0.12	—					
**pain intensity (NRS)**	0.36***	0.28	0.02	-0.002	0.05	0.36***	—				
**total sleep duration (h)**	0.04	0.20	0.14	-0.17	0.07	0.36***	0.27	—			
**PSQI total score**	0.21	0.12	0.12	0.21	0.06	0.45***	0.34***	-0.68*	—		
**AIS total score**	0.16	0.12	0.18	0.26	0.07	0.48***	0.36***	-0.48***	0.83***	—	
**FSS total score**	0.11	0.19	0.26	0.131	0.08	0.19	0.26	0.09	0.19	0.24	—

BMI, body mass index; PSQI, Pittsburgh Sleep Quality Index; AIS, Athens Insomnia Scale; FSS, Fatigue Severity Scale ***p <.001.

The regression model for AIS was statistically significant [*F*(13,78) = 5,32; *p* < 0,001], and the r-square value of 0.382 explained 38.2% of the variation. Significant predictors of AIS were the number of chronic diseases, anxiety disorders, and experiencing pain in the last 3 months (**Table 8**). If a patient declared more chronic diseases, the AIS score was higher (β = 0.23; p = 0.050). A higher AIS score was also obtained in patients with anxiety disorder (β = 0.71; p = 0.014) or borderline conditions (β = 0.49; p = 0.039) compared to those with no disorder. Patients who experienced pain for a few days in the past 3 months (β = -0.89; p < 0.001) or not at all (β = -0.73; p = 0.014) compared to those who experienced pain almost daily also had higher AIS values.

**Table 8:** Multiple linear regression model for AIS.

			95% *CI*				
Predictor	*B*	*SE*	*LL*	*UL*	*t*	*p*	β
Intercept	7.57	2.65	2.29	12.85	2.85	0.006	
Number of chronic diseases (n)	0.44	0.22	<-0.01	0.89	1.99	0.05	0.23
Diagnostic delay (years)	0	0.04	-0.09	0.09	-0.05	0.958	0
Infections in previous 3 months (n)	0.76	0.41	-0.04	1.57	1.89	0.063	0.18
Age (years)	0	0.04	-0.07	0.08	0.06	0.952	0.01
BMI (kg/m^2^)	0	0.1	-0.2	0.2	-0.02	0.988	0
Sex							
man – woman	1.12	0.94	-0.75	2.98	1.19	0.238	0.22
HADS, anxiety							
severe anxiety – no anxiety	3.64	1.44	0.77	6.51	2.53	0.014*	0.71
moderate anxiety – no anxiety	2.54	1.21	0.14	4.94	2.1	0.039*	0.49
HADS, depression							
severe depression – no depression	-1.37	1.95	-5.26	2.52	-0.7	0.485	-0.27
moderate depression – no depression	-0.34	1.3	-2.92	2.25	-0.26	0.795	-0.07
Frequency of general pain in previous 3 months							
for several days – almost everyday	-4.57	1.19	-6.94	-2.2	-3.84	< .001***	-0.89
for more than 30 days – almost everyday	-1.61	2.23	-6.05	2.83	-0.72	0.473	-0.31
not at all – almost everyday	-3.75	1.5	-6.73	-0.77	-2.5	0.014*	-0.73

AIS, Athens Insomnia Scale; HADS, Hospital Anxiety and Insomnia Scale; BMI, body mass index; *p<.05 *** p<.001.

We also created multiple linear regression model for PSQI (**Table 9**). This model was a good fit to the data [F(13,78) = 3.80; p < 0.001] and explained a total of 28.6% of the PSQI variance (adj. R2 = 0.286). Significant predictors of the PSQI score were the number of chronic diseases, anxiety disorders, and experiencing pain in the past 3 months. The PSQI total score was higher in patients with more chronic diseases (β = 0.26; p = 0.041). In addition, the PSQI total score was higher in patients with anxiety disorders than in those without anxiety disorders (β = 0.87; p = 0.005). Patients who experienced pain almost daily compared to those who had pain for a few days over 3 months had a higher PSQI total score (β = -0.51; p = 0.044).

**Table 9:** Multiple linear regression model for PSQI.

			95% *CI*				
Predictor	*B*	*SE*	*LL*	*UL*	*t*	*p*	β
Intercept	4.6	2.24	0.13	9.06	2.05	0.044	
Number of chronic diseases (n)	0.39	0.19	0.02	0.77	2.08	0.041*	0.26
Diagnostic delay (years)	-0.02	0.04	-0.1	0.05	-0.54	0.589	-0.05
Infections in previous 3 months (n)	0.47	0.34	-0.22	1.15	1.36	0.177	0.14
Age (years)	0.03	0.03	-0.04	0.09	0.88	0.38	0.1
BMI (kg/m^2^)	-0.02	0.08	-0.19	0.15	-0.21	0.833	-0.02
Sex							
man – woman	0.51	0.79	-1.07	2.09	0.65	0.52	0.13
HADS, anxiety							
severe anxiety – no anxiety	3.51	1.22	1.08	5.94	2.88	0.005**	0.87
moderate anxiety – no anxiety	1.57	1.02	-0.46	3.6	1.54	0.128	0.39
HADS, depression							
severe depression – no depression	-0.03	1.65	-3.32	3.26	-0.02	0.987	-0.01
moderate depression – no depression	0.47	1.1	-1.72	2.65	0.43	0.671	0.12
Frequency of general pain in previous 3 months							
for several days – almost everyday	-2.06	1.01	-4.06	-0.06	-2.05	0.044*	-0.51
for more than 30 days – almost everyday	-0.21	1.89	-3.97	3.54	-0.11	0.91	-0.05
not at all – almost everyday	-1.56	1.27	-4.08	0.96	-1.23	0.222	-0.39

PSQI, Pittsburgh Sleep Quality; HADS, Hospital Anxiety and Insomnia Scale; BMI, body mass index; * p <.05 **p <0.01.

### 3.3 Fatigue

Fatigue was reported in 52.2% (n=48) of patients. Patients with fatigue had a higher BMI and more comorbidities than those without fatigue (2.0 ± 2.7 vs. 1.0 ± 2.0; p=0.044) **(** **Table 4** **)**. The majority of patients with a diagnosis/history of neoplastic disease (n=10; 83.3%) experienced fatigue (p=0.029). Likewise, a large proportion of patients with autoimmune phenomena had fatigue (n=19, 70.4 % vs. n=8, 29.6%; p=0.038). Moreover, fatigue was frequent in patients who experienced general pain almost every day for 3 months prior to the study (n=21, 72.4%; p=0.002). Fatigue was also associated with insomnia (p=0.022) and poor sleep quality (p<0.001).

A higher percentage of fatigue was reported in women (n=30, 62.5%; p=0.06) and in patients with a longer diagnostic delay of PID than their counterparts (4.9 ± 6.9 vs. 9.3 ± 13.1 years; p=0.503); however, the differences were not statistically significant (**Tables 4**, **6**).

The regression model created for FSS was a good fit to the data [F(13,78) = 4.14; p < 0.001] and explained a total of 31% of the FSS variance (adj. R2 = 0.310). Sex, anxiety and depressive disorders, and experiencing pain in the last 3 months were significant predictors in the model (**Table 10**). There were higher FSS scores in women (β = -0.46; p = 0.021), in those with borderline anxiety disorders compared to those without disorders (β = 0.56; p = 0.027), in those with depressive disorders compared to those without depressive disorders (β = 0.85; p = 0.037), and in those who had pain almost every day compared to those who had pain for a few days over a 3-month period (β = -0.61; p = 0.015).

**Table 10:** Multiple linear regression model for FSS.

			95% *CI*				
Predictor	*B*	*SE*	*LL*	*UL*	*t*	*p*	β
Intercept	30.19	7.17	15.91	44.47	4.21	< .001	
Number of chronic diseases (n)	-0.69	0.61	-1.9	0.52	-1.14	0.258	-0.14
Diagnostic delay (years)	0.12	0.12	-0.13	0.36	0.96	0.342	0.09
Infections in previous 3 months (n)	-0.89	1.1	-3.07	1.3	-0.81	0.422	-0.08
Age (years)	0.04	0.1	-0.16	0.25	0.42	0.675	0.05
BMI (kg/m^2^)	0.36	0.27	-0.18	0.9	1.34	0.186	0.13
Sex							
man – woman	-6	2.54	-11.05	-0.95	-2.37	0.021*	-0.46
HADS. anxiety							
severe anxiety – no anxiety	6.97	3.9	-0.8	14.73	1.79	0.078	0.53
moderate anxiety – no anxiety	7.34	3.26	0.85	13.83	2.25	0.027*	0.56
HADS. depression							
severe depression – no depression	11.23	5.29	0.71	21.75	2.12	0.037*	0.85
moderate depression – no depression	6.82	3.51	-0.17	13.8	1.94	0.056	0.52
Frequency of general pain in previous 3 months							
for several days – almost everyday	-7.99	3.22	-14.4	-1.59	-2.48	>0.015*	-0.61
for more than 30 days – almost everyday	-3.51	6.04	-15.52	8.51	-0.58	0.563	-0.27
not at all – almost everyday	-5.11	4.05	-13.17	2.95	-1.26	0.211	-0.39

FSS, Fatigue Severity Scale; HADS, Hospital Anxiety and Insomnia Scale; BMI, body mass index; *p <.05.

### 3.4 Anxiety and depression

Among our patients, anxiety was more frequent than depression. Thirteen patients (14.1%) had a score ≥11, which indicates severe anxiety; 22 patients (23.9%) had ≥8 points, which indicates moderate anxiety; and 57 patients (62.0%) did not have anxiety. According to the HADS scale, 6 patients (6.5%) had severe depression with a score ≥11; these patients identified depression as a concomitant disease. Seventeen patients had moderate depression with a score ≥8 (18.5%), and 69 patients did not have depression (75%).

## 4 Discussion

To maintain good health, the recommended sleep duration is at least 7 hours per day (26). In our study approximately one-third of patients slept <7 hours, with the mean sleep duration of 6.99 ± 1.5 hours, compared to 7.7 hours in the general Polish population (27). Sleep latency is the length of time needed to fall asleep, and it is assumed to last <30 minutes (28). In our study, sleep latency was longer than half an hour in one-fourth of the patients (n=25; 27%).

Insomnia has been a concern in various studies, and its prevalence varies depending on the study population. A study conducted on adult individuals in Poland (n=47,924) revealed insomnia in 28.1% of women and 18.1% of men (29). However, subjective sleep quality and prevalence of insomnia in patients with PIDs have not been reported. To our knowledge, this is the first study to assess subjective sleep quality and the prevalence of insomnia among patients with PIDs.

Among the patients in our study, neither a particular disease nor type of treatment was associated with insomnia symptoms or subjective sleep quality. However, there is no scale to assess the severity of PIDs, and physicians use the number and severity of infections to determine disease control and potential exacerbations. Herein, patients with poor sleep quality had more infections in the previous 3 months than those with good sleep quality. Nevertheless, sleep duration was not associated with the number of infections (p=0.110). We could not determine whether our patients were prone to infections due to poor sleep quality or if their sleep was altered by ongoing infection. Sleep impairment may be an important factor that increases susceptibility to infections in patients with PIDs who have a primarily impaired immune system. Sanjay et al. (30) investigated the association between sleep duration and susceptibility to pneumonia in a prospective study. The researchers included 56,953 women without relevant comorbidities and a prior history of pneumonia and assessed sleep deprivation. They revealed that sleep durations ≤5 hour and ≥9 hours were associated with a higher risk of pneumonia compared with a sleep duration of 8 hours. In their experimental study, Cohen et al. (31) observed sleep efficiency and sleep duration in 153 healthy volunteers over 14 days. Subsequently, the researchers administered nasal drops with rhinovirus to the participants and observed them for 5 days following the development of a clinical cold. The authors proved that reduced sleep efficiency and shorter sleep duration prior to exposure to the virus were associated with lower resistance to respiratory illness.

More than half of patients (67.4%) in our study had chronic diseases other than PID. Almost half of them had lung disease, representing a frequent comorbidity of PID (32). Other frequent comorbidities were rheumatological, gastroenterological, and cardiovascular diseases, with each group comprising approximately one-fourth of patients. In our study, patients with poor sleep quality had significantly more chronic diseases than those with good sleep quality, as reported previously (18). Moreover, a higher number of comorbidities in our patients was related to poorer sleep quality. Basent et al. (33) recruited 5,878 individuals to investigate the association of common chronic diseases with sleep disorders and sleep quality. They found a significantly increased odds of poor sleep quality in patients with cardiac insufficiency, gallstone degenerative joint disease, and depression. A cohort study on individuals with COPD revealed poor subjective sleep quality in this group; interestingly, higher PSQI scores were associated with increased risk of COPD exacerbations during the follow-up (17). Matsuda et al. (34) assessed sleep quality using the PSQI during hospitalization for a broad spectrum of cardiovascular diseases. Almost half of the participants reported poor sleep quality.

In the regression model, we revealed that a higher number of comorbidities was a predictive factor for insomnia. Abad et al. (35) summarized that insomnia and unrefreshing sleep are common complaints in patients with rheumatologic disorders, such as, rheumatoid arthritis (RA), osteoarthritis, systemic lupus erythematosus (SLE), and Sjogren syndrome.

Momayyezi et al. (36) reported poor sleep quality in 69.3% of patients with cancer. In our study, 2 patients had ongoing cancer treatment, and both had poor sleep quality and insomnia. Among the cancer survivors, 7 (70.0%) had poor sleep quality, and 6 (60.0%) had insomnia. A study by Hammersen et al. (37) included 465 long-term lymphoma survivors in Germany and revealed poor sleep quality according to the PSQI in 224 (48.2%) patients. According to data obtained by Chen et al. (38), sleep quality measured by different methods, both subjective and objective, is affected in patients with cancers, particularly in those with lung, breast, gynecological, head, and neck cancers.

In our study, the incidence of autoimmune phenomena was 29.4% (n=27), which is comparable to that reported in other cohorts with PIDs (7). Patients with autoimmunity in our group had poorer sleep quality than those without autoimmune phenomena, and most of them had fatigue. Autoimmune diseases are also associated with sleep disorders (15). Many studies have investigated sleep quality in RA. They revealed that the vast majority of patients with RA have poor sleep quality, which may be associated with disease activity and ongoing inflammation (39). Likewise, patients with SLE report sleep disorders as frequent complaints with poorer sleep quality compared to the general population (40). Alterations in the immune system owing to sleep impairment may affect the course of autoimmune diseases. For instance, patients with Crohn’s disease have an increased risk of disease flair subsequent to sleep disorders (41). Furthermore, many patients with autoimmune diseases experience chronic pain (42), neuropathic, somatic, or visceral, depending on the particular diseases, which is an independent factor that diminishes sleep quality.

We asked patients about stressful events 3 months before the study. Patients with poor sleep quality, insomnia symptoms, and fatigue experience stressful events more frequently than those without. However, the differences were statistically relevant only for the PSQI score. A study conducted by Otsuka et al. in a Japanese population revealed a significant positive association between sleep disorders and high levels of stress. Furthermore, individuals with high stress are more prone to develop insomnia symptoms than those without (43).

Herein, our patients who experienced general pain almost every day for 3 months prior to the study had poor sleep quality, symptoms of insomnia, and fatigue more frequently than those without these disorders. The higher prevalence of pain was associated with poorer sleep quality and higher AIS total score (**Tables 8**, **9**). The connection between pain, sleep quality, and fatigue has been reported in many chronic diseases, including rheumatic disorders (44). Studies on RA and fibromyalgia have revealed that pain increases sleep disturbances (35). Patients with chronic pain have diminished subjective sleep quality compared with those without pain (45). Other studies have indicated that sleep disorders may initiate pain (16). Disrupted sleep also increases sensitivity to pain (41). This relationship has been proven in many studies, but its direction remains unclear. Many authors have highlighted the bidirectional relationship between pain and sleep disturbances (46–48). In some comprehensive analyses, sleep disturbances were considered a stronger predictor of the incidence of pain than pain as an inductor of sleep disturbances (44, 49). Furthermore, sleep disorders can result in fatigue (48) and depression (47).

In our study, 40.2% of patients were overweight and obese. BMI was associated with subjective sleep quality and fatigue in our study. Patients with poor sleep quality and fatigue had a higher BMI than those with good sleep quality and no fatigue **(** **Table 6**). In a study conducted by Fatima et al. (50), a considerably higher percentage of overweight and obesity was reported in patients with poor sleep quality than in those without.

Fatigue is a nonspecific symptom characterized by tiredness or inability to function due to a lack of energy (51), which cannot be restored by resting (52). Although fatigue appears in healthy individuals, it is more frequent in patients with chronic diseases (14), including various rheumatological diseases (15), neurological disorders (53), COPD (54), and cancers (52). Recent studies have shown that fatigue is independent of disease severity and activity (14).

In our study, fatigue was reported in 52.2% of patients. Hajjar et al. reported that among 2,537 patients with PIDs, 25.9% had fatigue, with the highest prevalence among patients with CVID (55).

Our linear regression model revealed higher prevalence of fatigue in patients with borderline anxiety disorders compared to those without disorders, and in patients with depressive disorders compared to those without depressive disorders (**Table 10**). Likewise, Bansal et al. investigated the prevalence of fatigue in patients with primary antibody deficiencies (PAD) and revealed a high frequency of fatigue in patients with PAD, which was correlated with the presence of anxiety and depression (56). Among our patients, anxiety was a relevant predictor of insomnia and poor sleep quality. Individuals with an anxiety disorder or borderline condition had higher AIS scores compared to those with no such a disorder (**Table 8**) (57).

## 5 Conclusions

Sleep quality, fatigue, pain, and primary immune disorders may give an impression of distinct medical problems; however, they appear to be connected in complex relationships. Therefore, they must be considered in a holistic model. As aforementioned, many factors may affect sleep quality, resulting in complex consequences. Our study, which is the first to address this issue, provides insight into the prevalence of insomnia, fatigue, and subjective sleep quality assessment in patients with PIDs. The data demonstrated that decreased sleep quality, insomnia symptoms, and fatigue are common in this group. There is a need for further studies to explain the determinants of poor sleep quality in this specific group of patients.

## 6 Limitations

Although this study was carefully planned, it has some limitations. First, subjective sleep quality was measured, which may have been affected by some errors. Although an objective measurement of sleep quality provides undeniable data, its use in daily clinical practice is limited owing to its high cost and time requirement. Second, we did not investigate other sleep disorders, except for the symptoms of insomnia and sleep quality. Third, the sample size of this study was small. In the pilot study, we recruited 92 patients from 4 clinical centers. Fourth, data concerning general health and the administration of medication were provided by the patients, and they were not subject to verification. We could not provide ICD codes for chronic diseases. This observational nature of the study may have created bias. Another limitation is that the majority of our patients had primary antibody deficiencies (PAD). Patients with other PID were the minority (n=4, 4.35%), and we could not analyze those patients separately.

## Data availability statement

The raw data supporting the conclusions of this article will be made available by the authors, without undue reservation.

## Ethics statement

The Independent Bioethics Commission for Research of the Medical University of Gdańsk approved the study (number: 422/2017). The patients/participants provided their written informed consent to participate in this study.

## Author contributions

KG and MZ designed the study with the support of KNS. KG and MZ prepared the first draft of the manuscript. This text was produced with equal contributions from both authors. KG, MZ, EW-S, AM-B, KN-B, and DS collected the data and performed literature searches. MZ and KG performed the statistical analyses. EW-S, AM-B, KN-B, ZZ, AH, and KN-S critically revised the manuscript for intellectual content. All authors contributed to the article and approved the submitted version.
